# Axillary Artery Injury in Proximal Humeral Fractures: Displacement Medial to the Coracoid on X-ray as a Risk Sign of Vascular Injury

**DOI:** 10.7759/cureus.105058

**Published:** 2026-03-11

**Authors:** Min Kyu Park, Neil Ashwood, Metwally Sayedahmed, Muhammad Bin Maidin, Andrew P Dekker, Amol Tambe

**Affiliations:** 1 Trauma and Orthopaedics, University of Leicester, Leicester, GBR; 2 Trauma and Orthopaedics, University Hospitals of Derby and Burton NHS Foundation Trust, Derby, GBR

**Keywords:** artery pseudoaneurysm, axillary artery injury, computed tomography angiography (cta), proximal humerus fracture, vascular repair

## Abstract

Axillary artery injury is a rare but serious complication of proximal humeral fractures that can threaten both life and limb. The artery lies in close proximity to the glenoid and humerus, making it susceptible to laceration, thrombosis, or pseudoaneurysm during trauma or surgical fixation. Medial translation of the humeral shaft under the coracoid on a radiograph may help surgeons predict arterial injury and prepare accordingly. A retrospective review of cases in a large UK shoulder unit covering a million patients identified five cases over the last three years where proximal humeral fractures were complicated by vascular injury during surgery. The arterial injuries ranged from axillary artery tears with catastrophic bleeding, branch lacerations, and the formation of pseudoaneurysms. Treatment required vascular surgical repair of tears, bypass grafting, and embolization through interventional radiology for a pseudoaneurysm. While there was no loss of life, brachial plexus injury occurred in the catastrophic bleed cases. Avoiding vessel tearing may have been achieved by first approaching the artery in cases where the humeral shaft lies beneath the coracoid. Early computed tomography angiography with a coordinated orthopedic-vascular multidisciplinary approach may facilitate preoperative recognition and management of this uncommon injury.

## Introduction

Proximal humeral fractures are the third most frequent osteoporotic fracture, with an incidence exceeding 1,500 cases per 100,000 person-years for women over 80 following low-energy falls [1]. Most cases are managed conservatively, but in 10% of cases with dislocation, damage to the head of the bone, or open injury, surgical intervention can improve outcomes. A final indication is neurovascular injury, which may be identified preoperatively through diminished pulses and neurological impairment. Occasionally, this type of injury can occur perioperatively or be unmasked at surgery when fracture reduction removes the tamponade of the axillary artery or vein, with distal ischemia avoided due to adequate collateral circulation post-injury.

The axillary artery is 11 cm in length and is enclosed in an axillary sheath, being divided anatomically into three parts by the pectoralis minor with six branches [2-4]. The artery is, on average, 10.3 mm from the glenoid, and this distance is halved by externally rotating the arm [2,3]. Translation of a fracture fragment with a sharp end risks lacerating the axillary artery and its branches.

Clinical signs of axillary artery injury (AAI) include an expanding axillary mass, diminished distal pulses, and significant ecchymosis in the axillary region. The authors postulate a further radiographic sign: medial translation of the humeral shaft fracture beyond a vertical line from the coracoid base. The authors retrospectively reviewed patients with axillary artery or its branches injured over the last three years, identifying five cases to assess the validity of this radiographic sign. This may enable identification of those injuries without clinical signs, facilitating preoperative imaging and the involvement of the vascular or interventional radiology team to reduce patient risk.

In the following case series, the patients did not undergo vascular imaging in the first two cases prior to surgery for their orthopedic issue, as vascular injury was not suspected based on the level of fracture displacement, and the vascular team had to attend expeditiously. The final three cases had imaging of expanding or unresolving hematomas, underscoring the importance of suspecting a vascular injury rather than a wound or infection at the fracture fixation site. Neurovascular examination was normal prior to surgery, so this did not raise suspicion of vascular injury. The patients were followed up for at least one year, with no vascular complications developing after the intervention.

## Case presentation

Case 1

A 73-year-old female with type 2 diabetes mellitus presented with an oblique fracture through the right neck of the proximal humerus following a simple fall from a standing height onto an outstretched arm with a normal clinical neurovascular examination. Given the displacement, surgery was planned to realign the fractures but also to look for artery involvement. At the time of surgery, the axillary artery was hitched on the end of the fracture and had to be isolated and then repaired primarily by the vascular team with restoration of circulation clinically and by visualization of the distal pulses using Doppler ultrasound.

**Figure 1:**
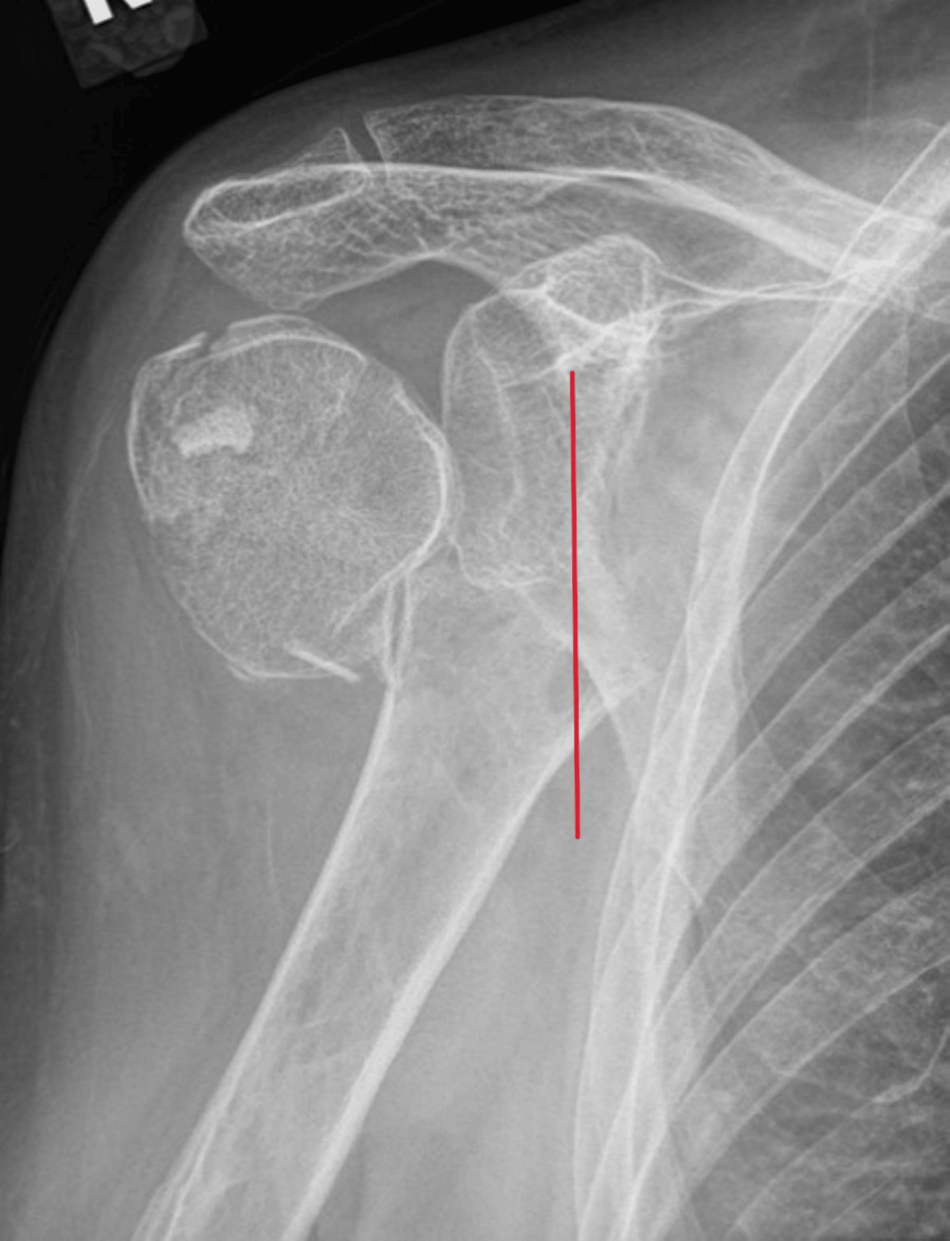
Preoperative anteroposterior radiograph of a 73-year-old female The line at risk shows significant medial displacement of the humeral shaft.

Radiographs of her humerus showed significant medial displacement of the distal shaft (Figure 1), with a fracture in the greater tuberosity. The fracture's position under the glenoid and in line with the coracoid makes AAI possible, prompting planning.

Open reduction and internal fixation with PHILOS plating was performed, and pulses were normal clinically and on Doppler ultrasound at the table. Angiography was not deemed necessary by the vascular surgeon.

**Figure 2:**
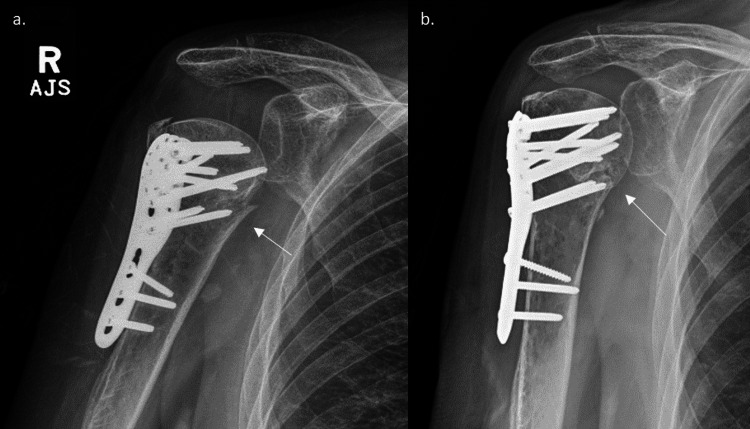
Postoperative anteroposterior radiographs at (a) one month postoperative and (b) seven months postoperative showing solid union of the fracture The white arrows show the approximate position of the arterial repair.

The postoperative course was uneventful, with shoulder radiographs at one and seven months after surgery showing satisfactory fracture union and restoration of vascular function (Figure 2). There were no clinical issues at one year, and the patient recovered fully.

Case 2

A 75-year-old female with osteoarthritis and hypertension presented with a right fragmented fracture of the proximal humerus following a fall. On initial inspection, the brachial and radial pulses were present, and the radial, ulnar, median, and axillary motor and sensory functions were intact on clinical testing.

**Figure 3:**
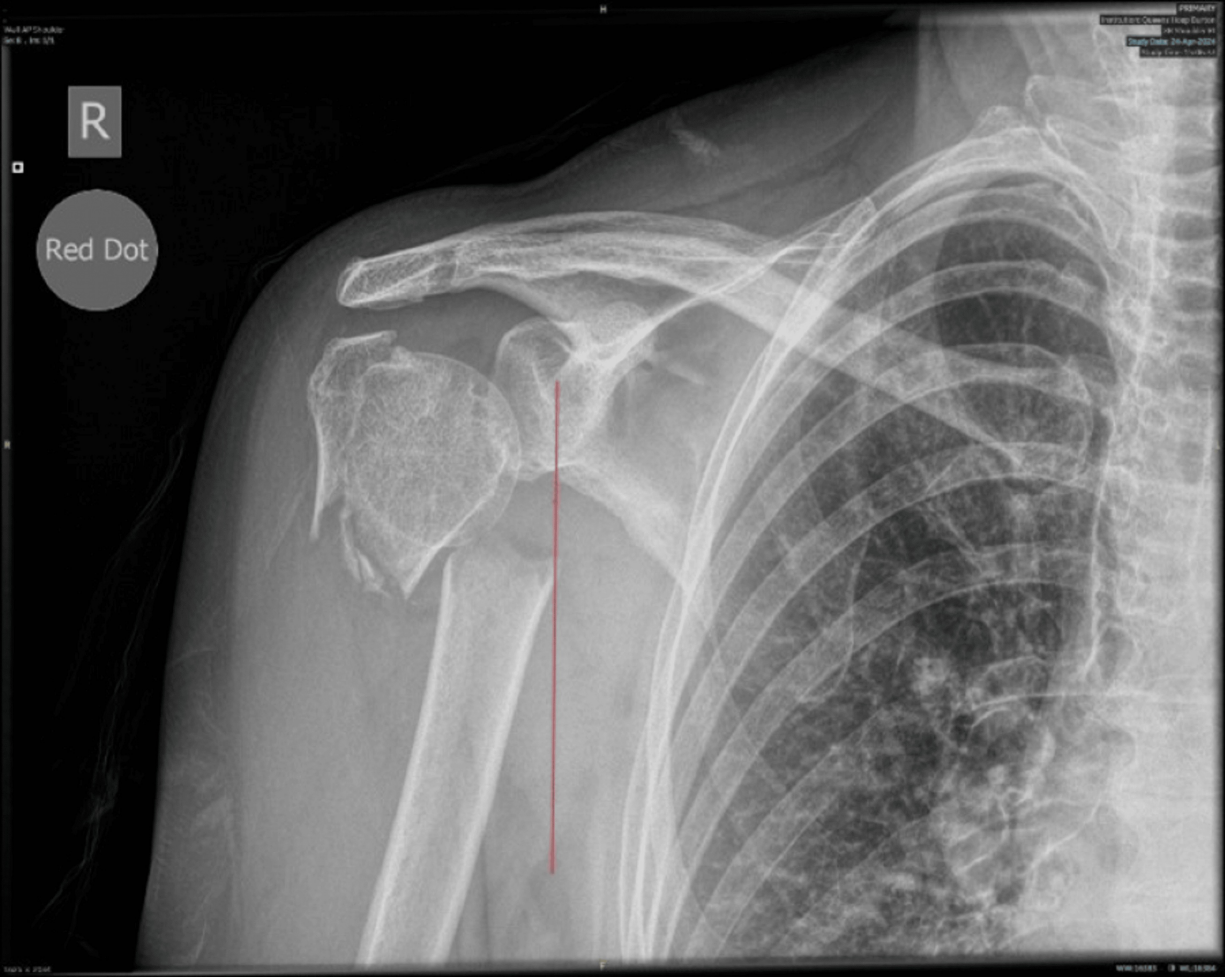
Preoperative radiographs demonstrating a fragmented transverse fracture of the proximal humerus with medial displacement of the shaft The line indicates the fracture is close to the axillary artery position.

Radiographs of the shoulder showed a transverse fracture of the humeral neck along with significant medial displacement and proximal migration of the humeral shaft (Figure 3).

**Figure 4:**
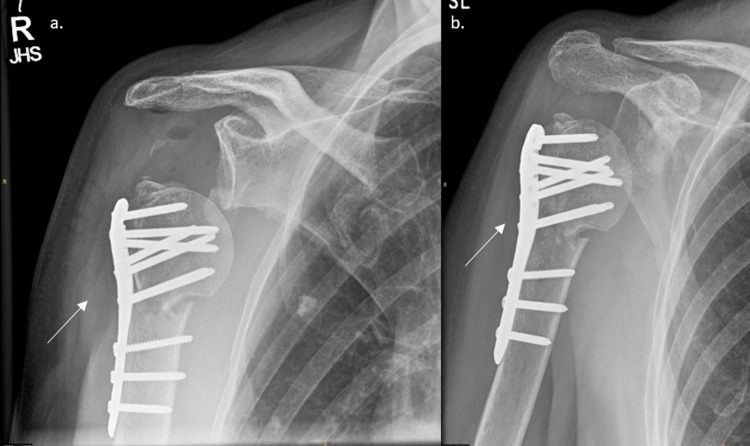
Postoperative radiographs taken at (a) two weeks and (b) six weeks postoperatively showing good fracture union The white arrows indicate the previous fracture.

Open reduction and internal fixation of the fracture were performed. At the time of surgery, a laceration of the axillary artery was noted due to the persistent bleeding at the fracture site, which was repaired directly by a vascular surgeon who had previously been alerted. Postoperative radiographs taken at two weeks and six weeks postoperatively showed satisfactory fracture union (Figure 4). The patient recovered well, with no ongoing neurovascular problems at one year; the vascular team did not indicate further vascular imaging.

Case 3

A 79-year-old male with chronic obstructive pulmonary disease and type 2 diabetes mellitus presented with right shoulder tenderness following a fall from a standing height onto the right shoulder.

**Figure 5:**
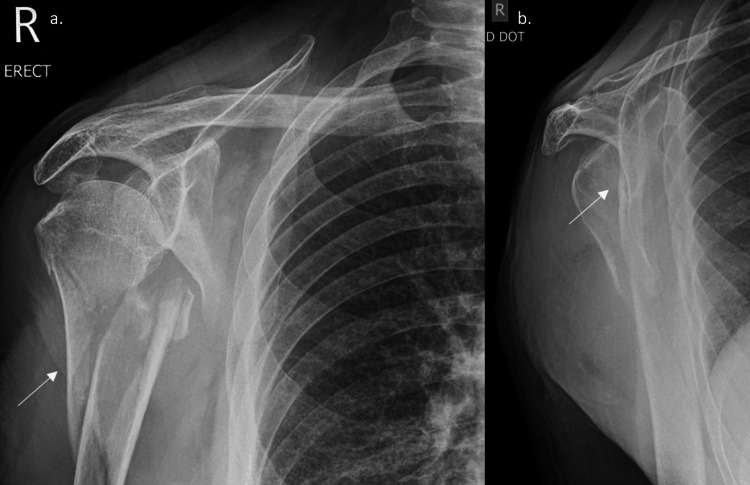
Initial radiographs showing a comminuted spiral fracture of the proximal humerus: (a) anteroposterior chest and (b) Y-view of the shoulder The white arrows indicate the fracture.

Radiographs of the shoulder showed a fragmented spiral fracture of the proximal humerus that extended to the humeral neck, along with anterior and medial displacement of the distal humeral shaft (Figure 5).

The patient was discharged with a trial of conservative treatment of a collar cuff and painkillers, with the hope that the hematoma would resolve as any leak settled. However, eight months later, he was presented with chronic shoulder pain, a healed fracture on radiographs, swelling of the right arm, and clinically a shoulder effusion, with signs of shock including hypoxia, tachycardia, and tachypnea.

**Figure 6:**
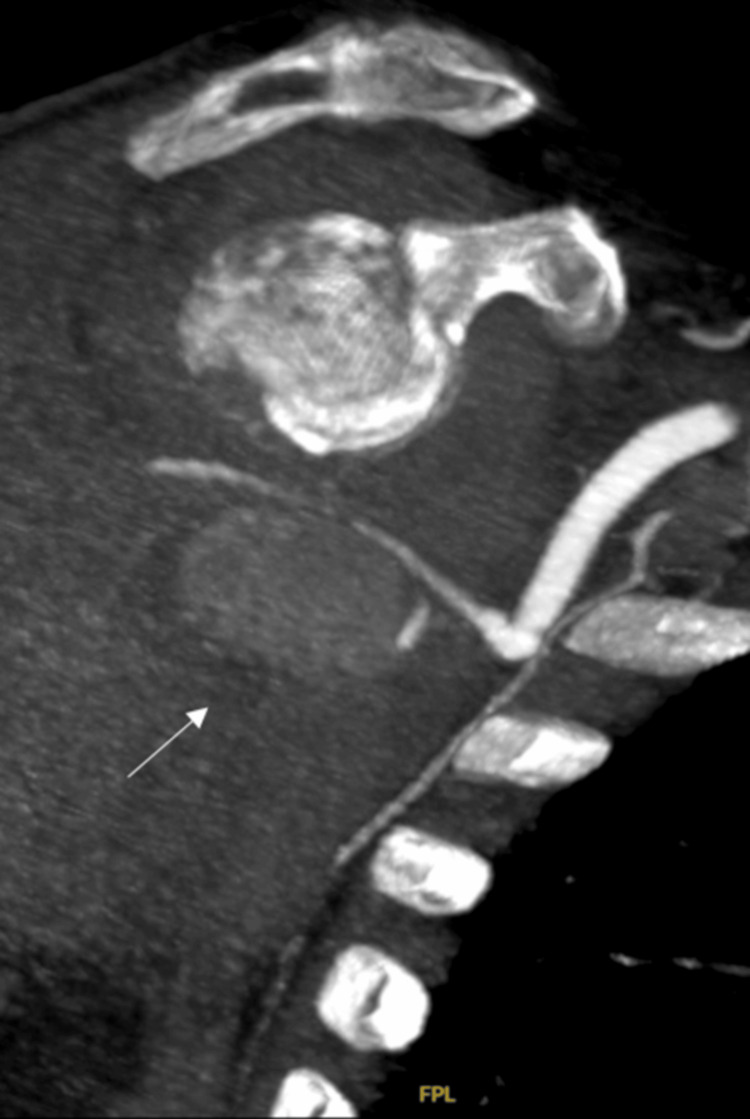
CTA showing a pseudoaneurysm of the posterior circumflex branch of the axillary artery The arrow indicates the pseudoaneurysm. CTA: computed tomography angiogram

Therapeutic shoulder arthroscopy and several washouts were performed because of concerns for septic arthritis, as the patient had a low-grade temperature of 37.5 degrees. However, four days after admission, the patient deteriorated with further bleeding, requiring a blood transfusion, and leading to further arm swelling. A consequent CT angiogram (CTA) showed a large soft tissue hematoma seen around the proximal humerus secondary to a pseudoaneurysm of the posterior humeral circumflex branch of the axillary artery (Figure 6) at the level of the previous fracture, which was in close proximity to the artery injury site. The CT did not indicate any other cause for the pseudoaneurysm, with no vasculitis or pre-existing vascular disease being evident.

**Figure 7:**
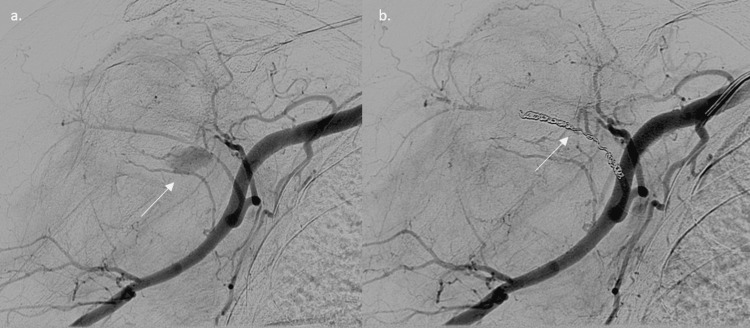
Angiography and embolization of a pseudoaneurysm of the axillary artery: (a) pre-embolization angiogram showing a pseudoaneurysm and (b) post-embolization showing complete exclusion of the pseudoaneurysm The white arrow indicates the pseudoaneurysm and embolization coil.

The pseudoaneurysm, 5 mm by 13 mm, fusiform, found 15 mm from the axillary artery within the posterior circumflex humoral artery, was embolized with fiber coils with the guidance of ultrasound. The post-coil angiogram shows satisfactory vessel occlusion with preservation of other branches (Figure 7). The hematoma resolved, and the soft tissue settled with intact pulses and neurovascular status seen clinically.

Case 4

**Figure 8:**
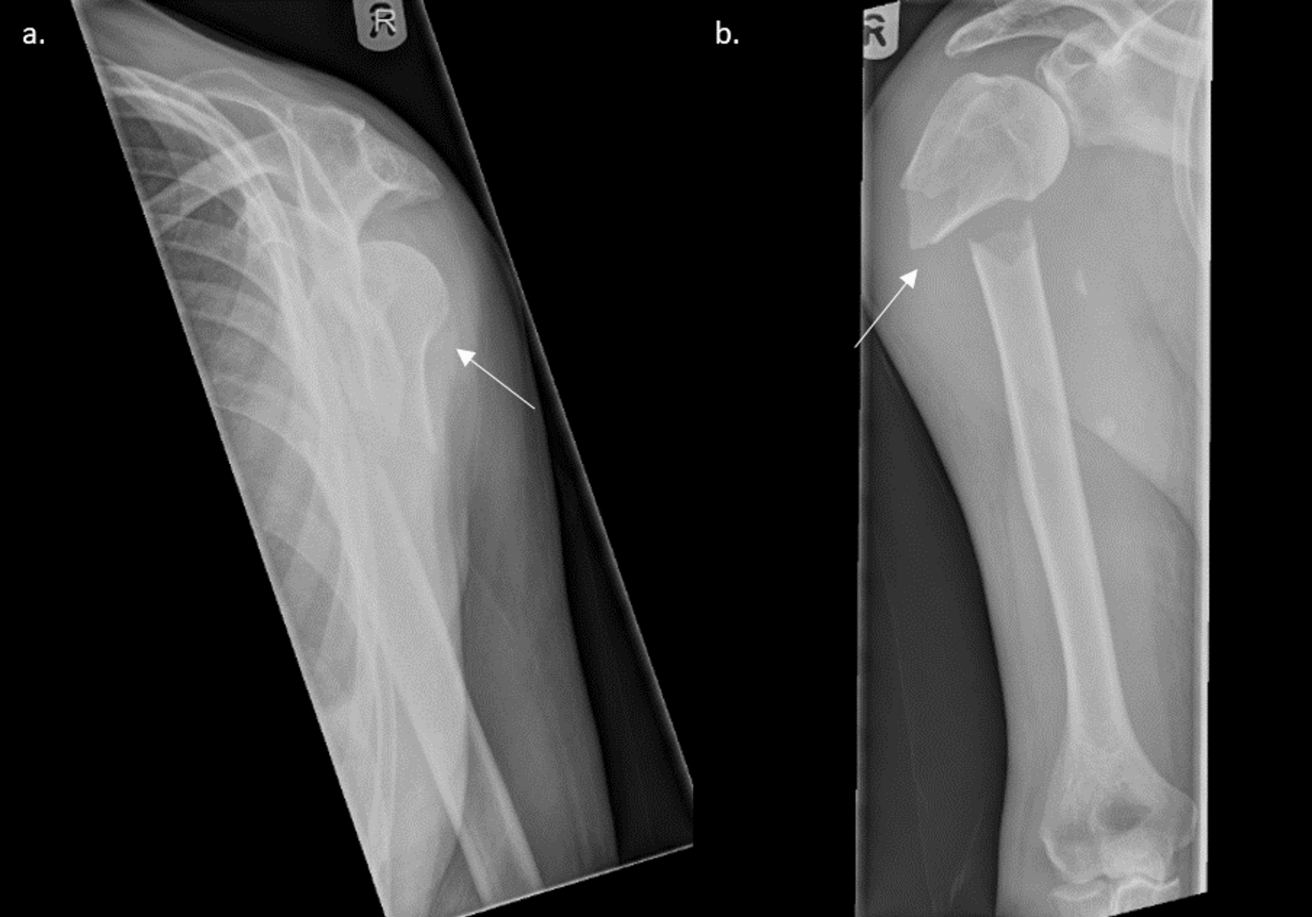
Preoperative anteroposterior radiographs of the right shoulder demonstrating a displaced proximal humeral fracture: (a) full-length shoulder view and (b) magnified view of the proximal humerus The white arrows indicate the fracture.

A 76-year-old male with progressive vascular dementia presented to the emergency department following agitation and aggression. During the hospital admission, the patient underwent an inpatient fall, sustaining an injury to the right humerus. Initially, the patient was treated with a sling only because the distal neurovascular structures appeared intact. However, the patient was non-compliant and developed extensive bruising, with repeat radiographs showing a displaced fracture (Figure 8).

**Figure 9:**
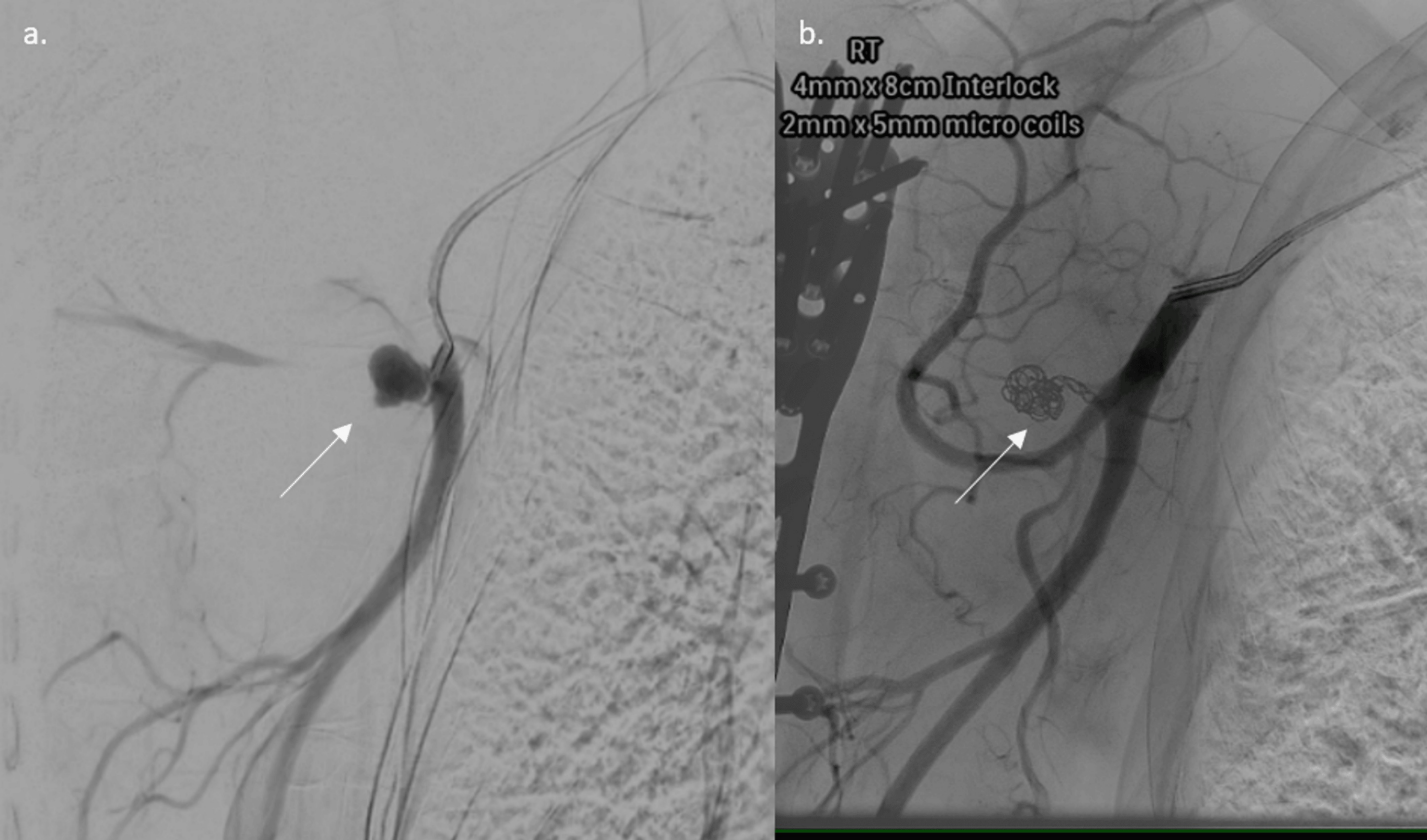
Angiography and embolization of a pseudoaneurysm: (a) pre-embolization angiogram showing a pseudoaneurysm and (b) post-embolization angiogram showing a complete embolization of the pseudoaneurysm The white arrow indicates the (a) pseudoaneurysm and (b) embolisation coil.

An open reduction and internal fixation with plating was performed, and during the procedure, a large hematoma was evacuated. It was felt to be secondary to the fracture hematoma by the operating surgeon. Postoperatively, the patient presented with profuse bleeding, with no neurological deficit in the arm, along with a limited range of motion. A repeat X-ray and CTA were done, revealing a pseudoaneurysm of a branch of the axillary artery. Persistence of the hematoma led to its subsequent embolization, with resolution of the collection (Figure 9). The patient's circulation, clinically and on ultrasound, remained intact. The patient's neurology was unaffected throughout, with a normal clinical examination.

Case 5

**Figure 10:**
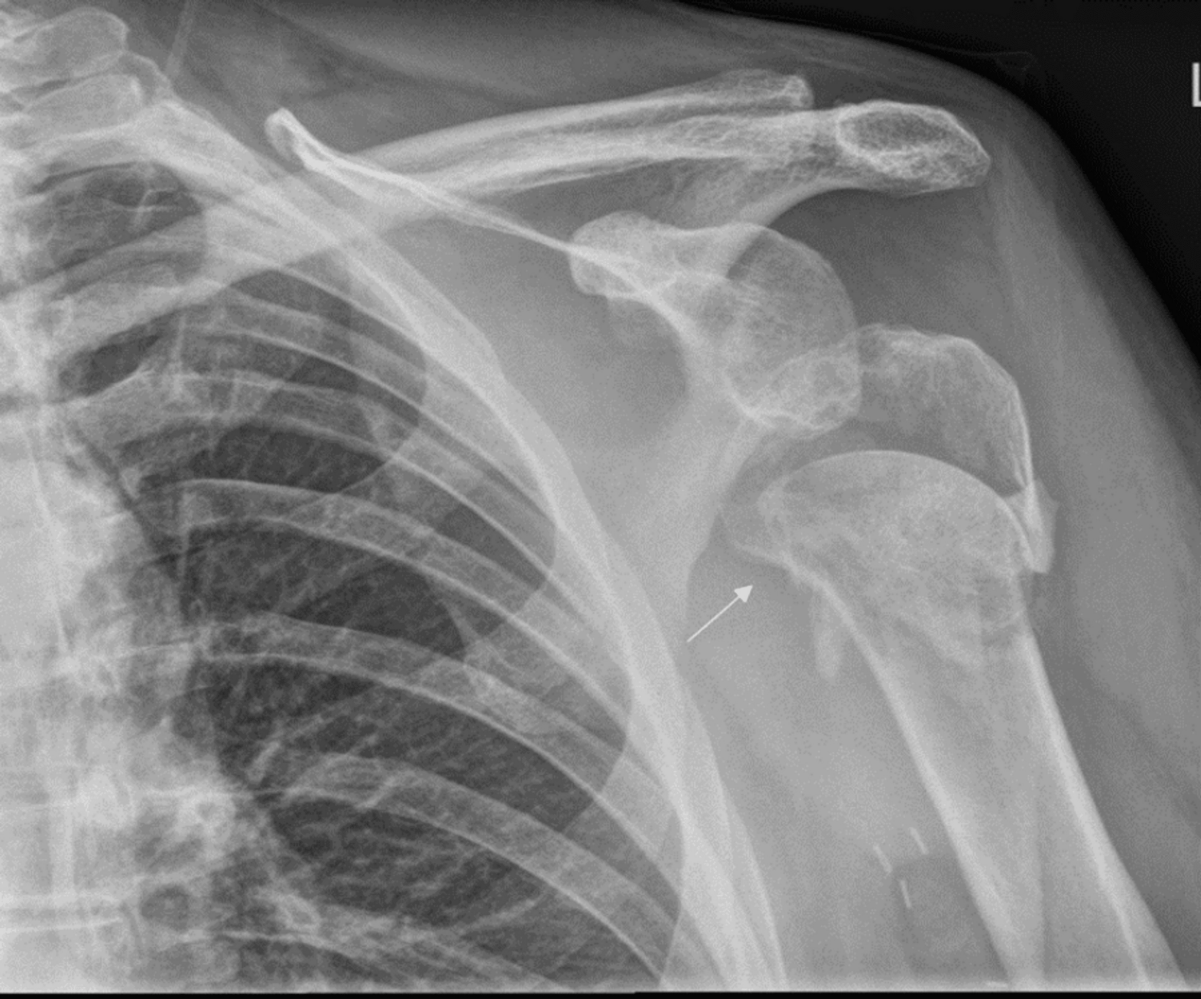
Preoperative anteroposterior radiograph of the left shoulder demonstrating a comminuted fracture-dislocation of the proximal humerus The white arrow indicates the fracture and dislocation.

A 74-year-old male presented to the emergency department following a fall from a ladder, sustaining facial injuries that included bilateral maxillary sinus and nasal bone fractures and a fragmented, displaced fracture dislocation of the left humeral head (Figure 10). The patient had normal neurological function and distal pulses on initial clinical examination.

**Figure 11:**
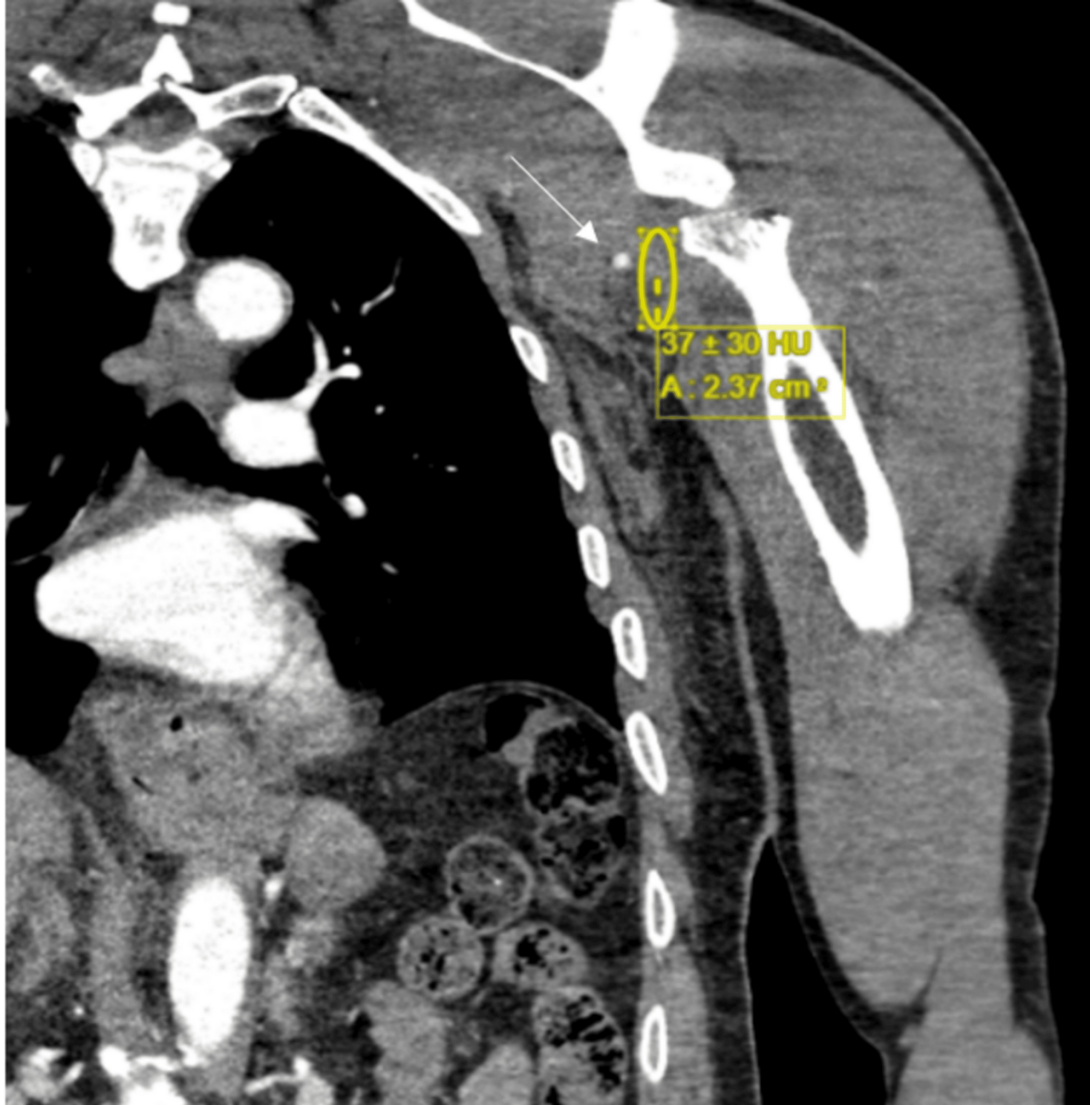
Coronal CTA of the left upper limb demonstrating axillary artery transection The white arrow indicates the position of the axillary artery. CTA: computed tomography angiogram

During admission, the patient developed acute axillary swelling, with reduced capillary refill time and absent pulses in the left hand. An urgent CTA was performed, which identified a transection of the axillary artery (Figure 11).

The patient underwent a reverse basilica vein patch to graft a defect within the artery, with good restoration of palpable pulses with a normal waveform on Doppler ultrasound. The decision was made to continue with the orthopedic procedure, and he had a reverse left cemented total shoulder replacement. Postoperatively, the patient achieved near-normal motion, with a Disability of Arm, Shoulder, and Hand (DASH) score of 14, a normal, palpable radial pulse, a warm, well-perfused hand, and no neurological disability.

These cases should alert clinicians to be more vigilant and to question the displacement of fractures and the presence of unresolving hematomas, to ensure timely, effective care for the fracture and any vascular injury, and to prevent delay or reoperation.

## Discussion

The synthesis of the case series and existing literature confirms that while AAI is a rare complication of proximal shoulder trauma, its catastrophic potential demands heightened clinical vigilance [1-3]. Suspicion arises when viewing all the imaging preoperatively, including X-rays and CT scans, since displacement of fragments close to the inferior glenoid edge on X-ray puts them close to the artery. The authors have used a vertical line from the coracoid, as it is easy to visualize, lies close to the danger area where the vessel lies, and appears significant in the cases reviewed. Indeed, the mean distance from the anteromedial tip of the coracoid process to the axillary artery is approximately 36.8 ± 6.1 mm, as measured by Nonthasaen et al. [5]. Some branches are even closer to the tip of the coracoid, with the thoracoacromial artery being even closer, with a mean distance of 31.89 mm from the coracoid tip [4-6]. This reference line using the coracoid seems to serve as a simple reminder of the radial artery's proximity preoperatively. Especially as abducting the arm in the beach chair position during reduction maneuvers shortens the distance from the tip of the coracoid to the artery to as little as 18 (+/- 5 mm). Surgeons should avoid blind reduction in cases with severe medial displacement and aggressive closed reduction maneuvers, as these risk intimal tears from shearing in calcified vessels in the elderly and complete arterial rupture from sharp fragments. The use of the coronoid as a reference point for potential fracture displacement leading to arterial involvement is one of the learning points from this article, as is the suspicion of vascular injury in the presence of a persistent hematoma. Future cases will be used to prospectively validate the predictability of this line.

Evolving diagnostic pathways: the necessity of CTA

The literature accurately notes that a preserved distal pulse due to rich collateral circulation is the most common diagnostic pitfall [6-8]. In the setting of a high-risk injury (subcoracoid fracture-dislocation, medial shaft displacement, or failed closed reduction), the decision to proceed directly to CTA may reduce the risk of this injury or at least alert the treating team to have the vascular team on standby.

CTA as Gold Standard

CTA has replaced conventional angiography as the gold standard for AAI in stable patients [7,8]. It not only identifies the site of rupture, occlusion, or pseudoaneurysm (Cases 3, 4, and 5) but also provides three-dimensional information essential for preoperative planning, defining the extent of soft-tissue hematoma, and aiding the orthopedic surgeon in identifying key neurovascular relationships prior to open reduction and internal fixation and vascular repair.

"Hard Signs Versus Soft Signs" Dilemma

While classic hard signs (absent pulse, expanding hematoma, distal ischemia) mandate immediate surgical exploration and repair, soft signs (pulse deficit, neurological deficit, fracture-dislocation) should trigger an immediate CTA, with medial displacement of a fracture spike beyond the coracoid base being recommended by the authors. Delayed diagnosis of pseudoaneurysms (Cases 3, 4) underscores AAI secondary to sharp fragments or iatrogenic injury, and closer follow-up of hematomas is required at weekly rather than monthly intervals to prevent problems from becoming indolent. Operating surgeons should be mindful of significant hematomas and consider an on-table angiogram where the source of the bleeding is not apparent and not controlled.

Management philosophy: the orthopedic-vascular protocol

The optimal management strategy is determined by the patient's hemodynamic status and the presence of limb ischemia.

Life-Threatening Ischemia/Active Bleeding (Case 5)

Vascular priority is absolute. A temporary shunt is placed to restore distal perfusion, followed by vascular repair (vein bypass graft, as done in Case 5) and then definitive orthopedic management (reverse total shoulder arthroplasty or fixation) to prevent repeat injury by the fracture fragments. Pressure and then clamping are facilitated surgically by removing the coracoid tip, which allows clearer visualization of the vessels. However, medial extension of the deltopectoral incision under the clavicle can facilitate proximal control with care to preserve the brachial plexus branches required.

Stable Patient With Contained Injury (Pseudoaneurysm/Branch Tear)

The increasing use of endovascular techniques is transforming care for AAI, especially in the elderly [9-11]. Coil embolization for branch injuries (e.g., posterior humeral circumflex artery, Cases 3, 4) and covered stents for main trunk injuries have lower morbidity and mortality rates compared to open repair in this high-risk, often frail elderly population [9-11]. Endovascular repair allows the orthopedic injury to be addressed either concurrently or in a planned, delayed fashion, without the complications associated with a large vascular exposure. Four washouts before identifying a pseudoaneurysm suggest a diagnostic delay, in which soft signs such as a persistent hematoma or wound complications fail to raise suspicion of ongoing vessel damage. A recommendation of this study is to suspect vascular injury when the initial radiographs show marked medial shaft displacement or, clinically, the patient has an ongoing hematoma.

Be vigilant for compartment syndrome in the forearm and hand following revascularization. If this is delayed, as reperfusion injury can lead to rapid swelling, and this in itself can compromise the microcirculation within the limb distally.

## Conclusions

In proximal humerus fractures, particularly those with significant medial displacement in the elderly, vascular status should be considered even with normal neurovascular examination. The need for pre-vascular imaging is a key recommendation from this series, and a non-resolving hematoma and X-ray displacement beyond the vertical line of the coracoid should trigger it. There must be a low threshold for arranging a CTA and coordination between orthopaedics, radiology, and vascular surgeons in a multidisciplinary team meeting. Vascular repair may be necessary at the same time as fracture fixation to ensure optimal functional limb salvage with care of the brachial plexus.

## References

[1] Walter N, Szymski D, Kurtz SM, Lowenberg DW, Alt V, Lau E, Rupp M (2023). Proximal humerus fractures - epidemiology, comparison of mortality rates after surgical versus non-surgical treatment, and analysis of risk factors based on Medicare registry data. Bone Joint Res.

[2] Yang K, Lee H, Choi IJ, Jeong W, Kim HT, Wei Q, Lee JH (2021). Topography and anatomical variations of the axillary artery. Biomed Res Int.

[3] Cotman SJ, Trinh TQ, Vincent S, Backes JR (2017). Proximal humerus fracture-dislocation with laceration of the axillary artery: a case report. Iowa Orthop J.

[4] Stone MA, Ihn HE, Gipsman AM, Iglesias B, Minneti M, Noorzad AS, Omid R (2021). Surgical anatomy of the axillary artery: clinical implications for open shoulder surgery. J Shoulder Elbow Surg.

[5] Nonthasaen P, Chaimongkhol T, Chobpenthai T, Mahakkanukrauh P (2025). Anatomical variations and surgical implications of axillary artery branches: an anatomical study of the coracoid process region. Anat Cell Biol.

[6] Modi CS, Nnene CO, Godsiff SP, Esler CN (2008). Axillary artery injury secondary to displaced proximal humeral fractures: a report of two cases. J Orthop Surg (Hong Kong).

[7] McFarland EG, Caicedo JC, Guitterez MI, Sherbondy PS, Kim TK (2001). The anatomic relationship of the brachial plexus and axillary artery to the glenoid. Implications for anterior shoulder surgery. Am J Sports Med.

[8] Hofman M, Grommes J, Krombach GA, Schmidt-Rohlfing B (2011). Vascular injury accompanying displaced proximal humeral fractures: two cases and a review of the literature. Emerg Med Int.

[9] McLaughlin JA, Light R, Lustrin I (1998). Axillary artery injury as a complication of proximal humerus fractures. J Shoulder Elbow Surg.

[10] Ripoll T, Fairag R, Bonomo I, Gastaud O, Psacharopulo D (2024). Axillary artery injuries associated with proximal humerus fractures: a literature review and a proposal of a novel multidisciplinary surgical approach. Vasc Endovascular Surg.

[11] Kriechling P, Whitefield R, Makaram NS, Brown ID, Mackenzie SP, Robinson CM (2024). Proximal humeral fractures with vascular compromise. Bone Joint J.

